# The Fire Fighter Cancer Cohort Study: Protocol for a Longitudinal Occupational Cohort Study

**DOI:** 10.2196/70522

**Published:** 2025-04-22

**Authors:** Jefferey L Burgess, Shawn C Beitel, Miriam M Calkins, Melissa A Furlong, Paola Louzado Feliciano, Jamie Kolar Gabriel, Casey Grant, Jaclyn M Goodrich, Judith M Graber, Olivia Healy, James Hollister, Jeff Hughes, Sara Jahnke, Krystal Kern, Frank A Leeb, Alberto J Caban-Martinez, Alexander C Mayer, Russell Osgood, Cynthia Porter, Sreenivasan Ranganathan, Heather M Stapleton, Natasha Schaefer Solle, Christine Toennis, Derek J Urwin, Michelle Valenti, John J Gulotta

**Affiliations:** 1 Department of Community, Environment and Policy Mel and Enid Zuckerman College of Public Health University of Arizona Tucson, AZ United States; 2 National Institute for Occupational Safety and Health Centers for Disease Control and Prevention Cincinnati, OH United States; 3 Department of Medicine State University of New York Medical University Syracuse, NY United States; 4 Los Angeles County Fire Department Los Angeles, CA United States; 5 D&S Research Associates and Engineers LLC Belmont, MA United States; 6 Department of Environmental Health Sciences University of Michigan School of Publich Health Ann Arbor, MI United States; 7 Department of Biostatistics and Epidemiology Rutgers the State University of New Jersey Piscataway, NJ United States; 8 Department of Epidemiology and Biostatistics Mel and Enid Zuckerman College of Public Health University of Arizona Tucson United States; 9 Orange County Fire Authority Irvine, CA United States; 10 Center for Fire, Rescue & EMS Health Research National Development and Research Institutes Leawood, KS United States; 11 First Responder Center for Excellence Crofton, MD United States; 12 Department of Public Health Sciences Miller School of Medicine University of Miami Miami, FL United States; 13 Firefighter Cancer Support Network Burbank, CA United States; 14 Fire Protection Research Foundation Quincy, MA United States; 15 Nicholas School of Environment Duke University Durham, NC United States; 16 Department of Chemistry & Biochemistry University of California at Los Angeles Los Angeles, CA United States; 17 Tucson Fire Department Tucson, AZ United States

**Keywords:** firefighter, cancer, prospective cohort study, biomonitoring, protocol

## Abstract

**Background:**

Firefighters are at an increased risk of cancer and other health conditions compared with the general population. However, the specific exposures and mechanisms contributing to these risks are not fully understood. This information is critical to formulate and test protective interventions.

**Objective:**

The purpose of the Fire Fighter Cancer Cohort Study (FFCCS) is to conduct community-engaged research with the fire service to advance the evaluation and reduction of firefighter exposures, along with understanding and mitigating effects leading to an increased risk of cancer and other health conditions. This involves establishing a long-term (>30 years) firefighter multicenter prospective cohort study.

**Methods:**

The structure of the FFCCS includes a fire service oversight and planning board to provide guidance and foster communication between researchers and fire organizations; a data coordinating center overseeing survey data collection and data management; an exposure assessment center working with quantitative exposure data to construct a firefighter job exposure matrix; and a biomarker analysis center, including a biorepository. Together, the centers evaluate the association between firefighter exposures and toxic health effects. Firefighter research liaisons are involved in all phases of the research. The FFCCS research design primarily uses a set of core and project-specific survey questions accompanied by a collection of biological samples (blood and urine) for the analysis of biomarkers of exposure and effect. Data and samples are collected upon entry into the study, with subsequent collection after eligible exposures, and at intervals (eg, 1-2 years) after enrollment. FFCCS data collection and analysis have been developed to evaluate unique exposures for specific firefighter groups; cancer risks; and end points in addition to cancer, such as reproductive outcomes. Recruitment is carried out with coordination from partnering fire departments and eligible participants, including active career and volunteer firefighters in the United States.

**Results:**

The FFCCS protocol development was first funded by the US Federal Emergency Management Agency in 2016, with enrollment beginning in February 2018. As of September 2024, >6200 participants from >275 departments across 31 states have enrolled, including recruit and incumbent firefighters. Biological samples have been analyzed for measures of exposure and effect. Specific groups enrolled in the FFCCS include career and volunteer structural firefighters, women firefighters, trainers, fire investigators, wildland firefighters, firefighters responding to wildland-urban interface fires, and airport firefighters. Peer-reviewed published results include measurement of exposures and the toxic effects of firefighting exposure. Whenever possible, research results are provided back to individual participants.

**Conclusions:**

The FFCCS is a unique, community-engaged, multicenter prospective cohort study focused on the fire service. Study results contribute to the evaluation of exposures, effects, and preventive interventions across multiple sectors of the US fire service, with broad implications nationally.

**International Registered Report Identifier (IRRID):**

DERR1-10.2196/70522

## Introduction

### Background

There are >1 million active career and volunteer firefighters in the United States [1]. Elevated cancer rates have been reported in US firefighters, including all cancer incidence and mortality; individual cancer sites, including but not limited to bladder, brain, colon and rectum, kidneys, lung and pleura, prostate, skin, and testis; as well as some hemopoietic cancers [2-4]. Occupational exposure as a firefighter is carcinogenic to humans, as determined by the International Agency for Research on Cancer (IARC) of the World Health Organization, based on sufficient evidence in humans for mesothelioma and bladder cancer [5]. While the IARC determination applies to all types of firefighters, most research supporting this determination has focused on career male municipal or structural firefighters. Far less information is available on other firefighter groups, including but not limited to volunteers, women as well as ethnic and racial groups underrepresented in the fire service, aircraft rescue and firefighting members, wildland firefighters, those responding to wildland-urban interface (WUI) fires, trainers, and fire cause investigators. Firefighting has also been associated with adverse reproductive effects. Specifically, male firefighters have been reported to have an increased risk of infertility [6], as well as an increased risk for birth defects in their children [7] and sperm abnormalities [8]. Female firefighters are at an increased risk of miscarriage [9], preterm birth [10], and reduced reproductive reserve [11].

Firefighters are exposed to numerous known, probable, and possible human carcinogens, including combustion products from fires (eg, benzene, polycyclic aromatic hydrocarbons [PAHs], and formaldehyde), asbestos, diesel exhaust, per- and polyfluoroalkyl substances (PFAS), flame retardants, and other hazards [12]. Exposure to some contaminants, such as PAHs, can occur both through inhalation and dermal exposure [13]. Night shift work, which is common among firefighters, has been determined as a probable human carcinogen [12]. However, we currently do not understand which individual exposures are responsible for cancer in firefighters, the mechanisms by which these exposures cause cancer, or the effective means of reducing exposures. As cancer has a long latency period, biomarkers that can measure the effects of carcinogen exposure are also needed well before the development of cancer, when interventions to prevent the disease could be effective. Furthermore, the IARC evaluation determined that there is strong evidence for 5 toxic mechanisms, also termed key characteristics of carcinogens, resulting from occupational exposure as a firefighter, including genotoxicity, epigenetic changes, oxidative stress, chronic inflammation, and modulation of receptor-mediated effects [12].

Because most published studies on firefighter exposures and effects have been based on predominantly male career firefighters, the cancer risk for other firefighter groups has not been adequately studied. Of the 1,041,200 estimated firefighters in the United States, 676,900 (65.01%) are volunteers [14], but information on the specific cancer risk of US volunteer firefighters is limited. The extent of cancer risk for wildland firefighters is also not known, despite the presence of known carcinogens in wood smoke [15], and exposure to wood smoke and combustion of biomass is associated with an increased cancer incidence in animal and human studies [16,17]. The limited available studies of US women firefighters suggest that career women firefighters are at an increased risk of cancer compared with the general population. A study of women career firefighters in Florida reported a 63% increase in overall cancer incidence as well as increases in cervical and thyroid cancers and Hodgkin disease compared to the general population [18]. Daniels et al [2] reported significant increases in bladder cancer incidence and mortality in women firefighters based on a few cases, as well as nonsignificant increases in breast cancer and overall cancer incidence. Published information on cancer rates for understudied racial and ethnic groups is also limited. In a study of California firefighters, cancers of the tongue, testis, and bladder; non-Hodgkin lymphoma; chronic lymphocytic leukemia; and chronic myelogenous leukemia were significantly elevated among understudied firefighter groups, predominantly Hispanic or Black (but not non-Hispanic White) firefighters compared to the general population [3].

### Objectives

The Fire Fighter Cancer Cohort Study (FFCCS) is designed to address the distinctive challenges of conducting research in the fire service because it is a community-engaged partnership of academic and governmental research centers with the fire service. In community-engaged research, community representatives and researchers share all aspects of the research process, including co‐learning and reciprocal transfer of expertise, shared decision-making, and mutual ownership of the processes and products of the research [19]. Community-engaged research with firefighters is critical to not only answer their research questions but also to effectively share this information with the broader fire service community and inform actions to protect their health. Furthermore, the prospective nature of the FFCCS allows for the collection of survey data and biological samples before the development of disease. The goal of the FFCCS is to work with the fire service to advance firefighter cancer control and prevention, as well as evaluation and prevention of other health conditions. The primary and secondary objectives of the FFCCS are listed in Textbox 1.

**Primary and secondary objectives of the Fire Fighter Cancer Cohort Study.**

**Primary objectives**

- Measure acute and chronic exposures experienced by firefighters
- Create a firefighter job exposure matrix
- Measure biomarkers of effect, including but not limited to epigenetic changes (eg, DNA methylation and microRNA expression)
- Compare exposures and effects across firefighter groups, including women; men; understudied ethnic and racial groups; volunteer, career, structural, wildland, and wildland-urban interface firefighters; trainers; investigators; and airport firefighters
- Communicate information regarding individual and aggregate study results to firefighter participants and the fire service community
- Identify characteristics of firefighters at increased risk for cancer
- Evaluate the effectiveness of exposure reduction and disease prevention interventions and communicate effective exposure mitigation approaches

**Secondary objectives**

- Evaluate the risk for reproductive health, mental health conditions, and other health conditions prioritized by the fire service
- Characterize noncarcinogenic exposures

## Methods

### Study Design

The FFCCS is a community-engaged prospective multicenter occupational cohort, enrolling incumbent firefighters at various points throughout their careers, as well as recruiting firefighters during their fire academy training, generally before their live-fire training. Most volunteer firefighters in the FFCCS were enrolled as part of the Firefighter Cancer Assessment and Prevention Study (CAPS), in collaboration with the FFCCS, with some differences in study procedures. CAPS enrolled participants from 41 US volunteer or combination (both volunteer and career) fire departments in Connecticut, Illinois, Kansas, Maryland, Maine, Missouri, New Jersey, Tennessee, and Washington. Participants enrolled through CAPS have been incorporated into the FFCCS for follow-up surveys and biological sample collection.

### Ethical Considerations

The FFCCS protocol was initially reviewed and approved by the University of Miami Institutional Review Board in 2017 (ethics approval number STUDY00002192). In October 2023, the reviewing institutional review board (IRB) was transferred to the University of Arizona. Academic and government partners have established reliance agreements with the reviewing IRB of record. The US Department of Homeland Security Compliance Assurance Program Office also reviewed and approved the projects funded by the US Federal Emergency Management Agency. Participants provided informed consent electronically through a REDCap (Research Electronic Data Capture; Vanderbilt University) study database system, with some using hard copy consent forms in situations where no internet access was available. FFCCS research personnel ensure that participants understand key study activities, data confidentiality, and risks before providing consent. FFCCS research personnel then countersign to confirm the consent. CAPS uses slightly modified consent procedures to allow researchers to obtain informed consent electronically without countersigning before the initial data collection event. Depending on the research project, participants are provided personalized results from analysis of their collected specimens.

### FFCCS Organizational Structure

**Figure 1:**
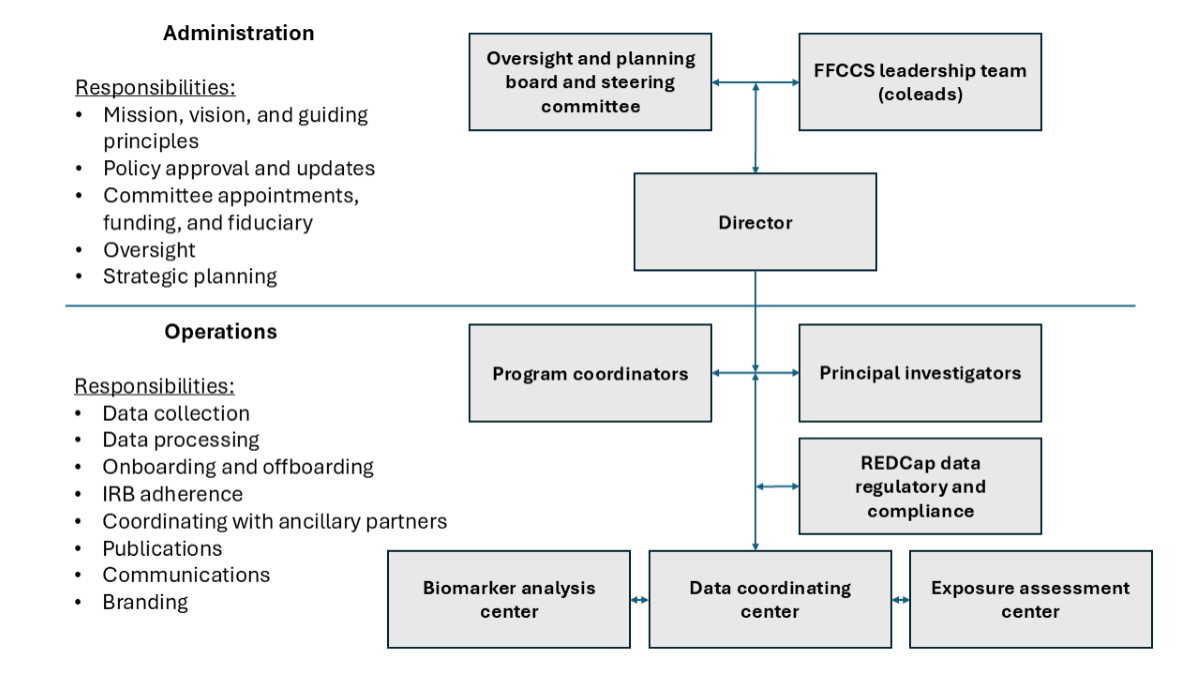
The Fire Fighter Cancer Cohort Study’s (FFCCS) organizational structure. IRB: institutional review board; REDCap: Research Electronic Data Capture.

The FFCCS is organized with administrative and operational units (Figure 1). The oversight and planning board (OPB) is composed of representatives from national fire service organizations and individual firefighters with expertise in active research projects who review current and new research projects under consideration, FFCCS policies, and FFCCS committee appointments. An OPB steering committee composed of a limited number of OPB members takes a more active role in FFCCS operations, including participation in the FFCCS leadership team. The FFCCS leadership team, called coleads, is composed of FFCCS center directors, individual research program principal investigators (PIs), and select OPB steering committee members. The coleads are responsible for making research operational decisions, with guidance from the OPB and OPB steering committee members. The daily operation of the overall FFCCS is run by the director. Individual FFCCS projects are run by their PIs and program coordinators. FFCCS committees (eg, publication committee) are established on an as-needed basis and can include coleads, other research faculty staff, and OPB members. The REDCap data manager and regulatory and compliance manager assist all PIs and report to the FFCCS director.

There are 3 FFCCS centers. The data coordination center designs and updates standardized participant survey data collection tools and analysis protocols to address the short- and long-term study objectives, as well as to facilitate any future linkages with long-term outcome data, including cancer development and mortality. The data coordination center also manages the survey data and oversees the process of internal and external data requests. The exposure assessment center collects and combines quantitative biomarkers of exposure measurements with information gleaned from self-reported characteristics of exposure (eg, job history, job task, and personal protective equipment) for the development of a job exposure matrix (JEM). These data will provide improved occupational exposure data for comparison with biomarkers of effect and eventual cancer outcomes. The biomarker analysis center provides a biorepository for blood and urine samples and carries out studies of biomarkers of exposure and effect, including epigenetic markers, comparing firefighters with a range of acute and cumulative exposures.

### Community Engagement and Policy

For many career and a few volunteer partner fire departments, ≥1 firefighter research liaison selected by the department coordinates FFCCS participant enrollment and follow-up. Firefighter liaisons serve a critical role in community collaboration and are vital to the success of the study. At least 1 liaison is onboarded to the IRB-approved protocol following the completion of a shortened human participants training course and receipt of a signed permission form from the department. Only IRB-approved liaisons have access to information on who has enrolled in their departments for which they are liaising and can assist with contacting participants to coordinate follow-up collections and complete study materials as needed. Study personnel, primarily study coordinators at academic institutions, provide frequent updates to department liaisons with information on study goals, progress, and next steps. Department liaisons are encouraged to engage with the academic team to provide feedback on study design and implementation. Department liaisons are often asked to review study materials, such as surveys and results reporting formats, to provide feedback and ensure that study materials are relevant to the fire service. To help ensure community engagement, fire department liaisons are notified before study materials (ie, participant results and newsletters) are sent to participants.

In addition, fire department liaisons are provided with information on study results before academic publication and dissemination. Study liaisons may be involved in manuscript development and study presentations. Furthermore, department liaisons provide firsthand knowledge of the fire service and are vital for identifying relevant research questions and ensuring that the research is aligned with the fire service. For some departments, such as volunteer departments participating in CAPS, research liaisons primarily support general communications and organizing study sessions rather than participant-specific activities. In these cases, research liaisons are not required to complete human participants research training. Working with firefighter liaisons through these avenues ensures consistent input from community partners and reinforces collaboration within the fire service.

### Individual Research Projects

The FFCCS is composed of individual, separately funded research projects with overlapping periods of performance (Table 1). These projects are based on research needs within the fire service, with fire departments requesting specific research studies from academic partners or vice versa. Academic institutions participating in the FFCCS have included Colorado State University; Duke University; National Development & Research Institutes (Kansas City); Rutgers, the State University of New Jersey; Tennessee Technological University; the University of Arizona; University of California at Los Angeles; University of Miami; University of Michigan; University of Washington; and Wildfire Conservancy. Government institutions directly involved in the FFCCS or providing analytical support have included the National Institute for Occupational Safety and Health (NIOSH) within the Centers for Disease Control and Prevention (CDC), the National Center for Environmental Health (NCEH) within the CDC, and the New Jersey Department of Health Public Health and Environmental Laboratories.

**Table 1:** Individual Fire Fighter Cancer Cohort Study (FFCCS) projects. Additional funding has been provided by individual fire departments, the International Association of Fire Fighters, and the National Institute of Environmental Health Sciences (NIEHS).

Project	Initial funding	Funding agency	Objectives
Pre-FFCCS	2015	FEMA^a^	Developed the community-engaged protocols for the FFCCS in partnership with the Tucson Fire Department, evaluated exposure reduction interventions, and established the association of epigenetic markers with cumulative firefighter exposures
Framework	2016	FEMA	Established the multicenter IRB^b^-approved protocol; baseline survey; partnership of academic, government, and fire service partners; as well as the OPB^c^, DCC^d^, BAC^e^, and EAC^f^
Expansion	2018	FEMA	Added more fire departments and focused on evaluating exposures and epigenetic markers of toxicity and cancer risk in firefighter trainers, fire investigators, and volunteer firefighters
PFAS^g^	2019	FEMA	Evaluated PFAS exposure in aircraft rescue and firefighting members as well as PFAS exposure from wearing new gear and responding to fires
NIOSH^h^ WUI^i^	2019	NIOSH within CDC^j^	Evaluated exposures and epigenetic effects of these exposures in firefighters from 2 California County fire departments during the campaign WUI fires
Women project (2 projects)	2020 and 2022	FEMA	Addressing priority research areas requested by women firefighters, including cancer risk, reproductive health, and stress
Volunteers and CAPS^k^ (2 projects)	2020 and 2022	FEMA	Added volunteer and combination fire departments and focused on evaluating environmental exposures (PFAS) and cancer risk factors and behaviors, as well as cancer risk reduction
Wildland (2 projects)	2021 and 2023	FEMA	Added both wildland and WUI exposure monitoring, evaluation of carcinogenic effects, and testing of exposure reduction interventions
Arizona PFAS	2023	Arizona Board of Regents	Evaluating serum levels of PFAS in firefighters and opportunities for linked randomized controlled trials designed to reduce serum levels of PFAS and improve epigenetic age
High risk	2023	FEMA	Focused on evaluating the effects of firefighter exposure to high-risk, low-frequency incidents, such as the train derailment and fire in East Palestine, Ohio, and the wildfires in Maui, Hawaii
North Carolina PFAS	2024	State of North Carolina	Enrolling additional North Carolina firefighters into the FFCCS and evaluating their PFAS exposure and health effects
NIEHS WUI	2024	NIEHS	Enrolling Los Angeles County Fire Department and Orange County Fire Authority firefighters likely to respond to WUI fires, evaluating exposures and exposure reduction interventions

^a^FEMA: Federal Emergency Management Agency.

^b^IRB: institutional review board.

^c^OPB: oversight and planning board.

^d^DCC: data coordinating center.

^e^BAC: biomarker analysis center.

^f^EAC: exposure assessment center.

^g^PFAS: per- and polyfluoroalkyl substances.

^h^NIOSH: National Institute for Occupational Safety and Health.

^i^WUI: wildland-urban interface.

^j^CDC: Centers for Disease Control and Prevention.

^k^CAPS: Firefighter Cancer Assessment and Prevention Study.

### Study Population

#### Overview

The enrollment goal for the FFCCS is 10,000 firefighters, with the representation of major firefighter employment types (eg, career and volunteer), response activities (eg, structural and wildland firefighters, those responding to WUI fires, aircraft rescue and firefighting members, trainers, and fire investigators), and geographic (urban, suburban, rural, and state) and demographic (sex, race, and ethnicity) groups across the United States. The study duration is planned for a minimum of 30 years, given the rolling cohort entry and latency period between the first cancer-related mutation and diagnosis of cancer, which can exceed 25 years for some forms of cancer [20].

The sample size of 10,000 firefighters will permit the evaluation of the potential for biomarkers to predict cancer outcomes and provide adequate power to evaluate the incidence of more common cancers, such as breast and prostate cancers. With a proposed sample size of 2000 female firefighters and a new case rate of breast cancer in the general population of 129.4 per 100,000 women each year [21], we estimate that we will have 80% statistical power to detect a 30% increase in breast cancer among female firefighters followed for >30 years compared with age-, race-, and calendar period–standardized rates in the US general population. For context, breast cancer in female individuals occurred at a (nonstatistically significant) 45% increased rate in a previous retrospective study of career female firefighters, with the US population referent [2]. For prostate cancer, with a proposed sample size of 2000 firefighters from understudied (predominantly Hispanic and Black) ethnic and racial groups, compared with >3700 non-Hispanic White career firefighters, and assuming a 5.2% cumulative 30-year incidence in all firefighters [2], we anticipate having 80% power to detect a 32% difference in prostate cancer rates between the 2 groups*.* A previous study found a 2.42-fold increase in prostate cancer odds ratio in understudied ethnic and racial group firefighters and a 1.4-fold increase in non-Hispanic White firefighters compared to the general population [3].

#### Recruitment and Enrollment

Our recruitment strategy generally works through individual fire departments; for specific populations (eg, volunteer women firefighters), we may enroll individual firefighters without prior department contact. Department contact begins with discussions with fire department representatives, followed by discussions with department administration and, for career departments, union representatives. Signed letters of support are obtained before the start of enrollment. Recruitment materials, such as study flyers, may be shared by email through the department, and informational webinars may be provided if the department or union requests. For some studies, fire department liaisons may visit each fire station and shift to provide additional information, and a scheduler platform may be used for interested participants to sign up for enrollment days and times. The voluntary nature of participation in the FFCCS is emphasized repeatedly. All active-duty firefighters aged ≥18 years with potential for fireground exposure are eligible for participation. Enrollment typically occurs at central locations or stations identified by our fire service partners. At these events, study researchers and firefighter liaisons explain the study to participants before they provide informed consent using an electronic consent form or a paper consent form if needed as backup. Following consent procedures, participants complete the enrollment questionnaire and provide biological samples. For CAPS, participants complete the consent form and enrollment questionnaire before meeting with the study team for sample collection.

### Data Collection Methods

The FFCCS study design is based on the collection of 3 primary sources of data: information provided through surveys; measures of exposure and effect in biological samples; and departmental fire run data, when available. Additional data are collected for specific populations or projects as needed. Examples include exposures measured in air or passive silicone sampling devices.

#### Survey Data Collection

All FFCCS participants complete an enrollment survey. Subgroup-specific surveys may be used for enrollment and as needed subsequently, such as after biological sample collection, to capture information about firefighter subgroups. Annual follow-up surveys were rolled out to all CAPS participants starting in 2023 and are being sent to all FFCCS participants starting in 2024. The enrollment survey collects fire service and other occupational history and exposures, sociodemographic information, and cancer history and cancer-related risk factors. These risk factors include questions about health care access and cancer screening, mental health, behavioral factors (eg, physical activity, tobacco, and alcohol use), as well as height and weight to calculate BMI. The annual follow-up survey builds upon the enrollment survey, asking participants for changes in the aforementioned categories since enrollment, and provides an opportunity to recapture any missing data.

#### Biological Samples

Urine and blood are collected at enrollment and, when possible, approximately every 2 years (18-24 months) thereafter; through retirement from the fire service; or conclusion of the study (whichever comes first). They may also be collected for individual research projects at additional specified intervals. Additional biological samples collected differ based on the project.

Blood is collected using a butterfly needle by a trained phlebotomist. The blood tubes collected include 2 glass red-top Vacutainer tubes (supplier number: 366430, Beckton, Dickinson and Company); 1 plastic blue-top sodium citrate Vacuette tube (supplier number: 454332, Greiner Bio-01e); 1 or 2 plastic purple-top ethylenediaminetetraacetic acid (EDTA) Vacuette tubes (supplier number: 456002, Greiner Bio-01e), depending on the project; and 1 Tempus Blood RNA tube (supplier number: 4342792, Applied Biosystems). After collection, the serum tube is left for 1 hour at room temperature to allow clotting, and the other tubes are immediately stored at 4 °C. All blood tubes are shipped at 4 °C to the processing facility at the University of Arizona. Samples are stored at 4 °C until they are processed within 24 to 48 hours of collection. The Tempus blood is divided into two 4.5 ml aliquots, and 2.5 ml of whole blood is removed from the EDTA tube for storage. The remaining blood is centrifuged at 1300 revolutions per minute and 4 °C for 15 minutes. Two 0.5 ml aliquots of plasma are extracted from the separated EDTA blood, and the remaining packed cells are stored. Three 0.5 ml aliquots of plasma are extracted from the sodium citrate tubes. A total of 7 aliquots of serum are extracted from the glass Vacutainer tubes, ranging from 0.5 to 2.5 ml. All samples are documented and stored in the study biorepository at –80 °C.

Urine is collected by participants in polypropylene collection cups (supplier number: 01 0100, Thermo Scientific) and stored and transported on dry ice to the processing facility at the University of Arizona, NIOSH, or another research laboratory. Upon receipt at the laboratory, urine is stored at –80 °C until it is processed. All urine undergoes 1 freeze-thaw cycle before processing to maintain consistency. Urine is thawed in a shaking incubator at 25 °C and 100 revolutions per minute for 2 hours and then divided into six 1.8 ml aliquots and one 12 ml aliquot. The samples are documented and stored in the study biorepository at –80 °C.

### Exposure Assessment

#### Overall Approach

In a subset of many firefighter subgroups, we have collected quantitative exposure measurements using biological samples (eg, blood and urine), self-reported exposure data from exposure tracking applications (eg, type of response, use of personal protective equipment, and presence of smoke), characteristics of exposure (eg, job history and job title) from the enrollment survey, and incident records from participating fire departments. Over time, these data will be used to develop and validate a JEM to better understand the potential exposures for firefighters by job title (eg, captain, firefighter, or paramedic), employment type (eg, volunteer and career), and subgroup (eg, wildland firefighters, fire cause investigators, and trainers).

#### Exposure Assessment Biomonitoring

For JEM development, blood samples collected at enrollment into the study and approximately 1 year to 2 years later (when feasible) may be analyzed for brominated flame retardants and PFAS. In addition, firefighters’ self-collected urine samples at enrollment into the study and 3, 6, and 24 hours after up to 10 fire responses over a period of 2 years may be analyzed for urinary metabolites of PAHs. In some substudies, spot urine samples are collected at the beginning and end of a shift (eg, after training fires or after WUI fire responses). For some substudies (ie, WUI study), urine samples may also be analyzed for other compounds (eg, volatile organic compounds [VOCs], heavy metals, organophosphate esters, and PFAS).

#### Exposure Tracking Application Data and Fire Department Records

If fire department records are not available, self-exposure tracking applications used include the National Fire Operations Reporting System (NFORS) developed by the International Public Safety Data Institute and the Personal Exposure Reporter (PER) developed by the University of Miami. For all FFCCS structural fire department participants, when possible, we request a minimum records-based exposure dataset (Textbox 2) from their fire departments between baseline and follow-up collections. A similar minimum exposure dataset is being developed for wildland firefighters, adapted for their operations. For individual projects where departmental data are not easily obtained (eg, volunteer fire departments) or additional exposure information is needed (eg, fire cause investigators), the participating firefighters have also been asked to complete either the NFORS or PER exposure tracking application.

**Minimum dataset for structural fire department.**

- Participant identification number
- Date range (participant’s enrollment to specified follow-up date)
- For each type of fire within the date range
  Run number (if future information on a fire is needed)
  Date and time of call
  Type of fire (eg, structural and vehicle)
  Elapsed time on scene
  Rank
  If available, job assignment on the fire (eg, rapid intervention crew)
- Total number of emergency medical service calls during the date range

#### Industrial Hygiene Evaluation

For some substudies (eg, trainers in the expansion study), industrial hygienists are present during fire responses and conduct environmental monitoring, such as area and personal air sampling. Exhaled breath samples are also collected before and immediately after fire responses. Air samples are analyzed for compounds that are also measured in urine (eg, PAHs and VOCs) and exhaled breath (ie, VOCs) to allow for comparisons between airborne exposures and internal dose [22].

Furthermore, passive silicone samplers (eg, wristbands) may be worn during some substudy activities, such as wildland fire response, and analyzed for exposures that are measured in the air or biological samples (eg, PAHs and brominated flame retardants) [23-25].

#### JEM Creation

Incident records from fire departments and self-reported NFORS or PER records will be modeled against quantitative exposure data (ie, urinary and blood concentrations) to identify the primary determinants of the systemic exposures. This allows for an evaluation of the association of fireground and emergency medical service exposures with measured exposure biomarkers (eg, serum PFAS or brominated flame-retardant measurements) and outcomes (ie, epigenetic markers) [26-28] and can also be used to estimate exposures using the exposure tracking application data. Within the JEM, exposures among the different firefighter subgroups and job titles will be compared to identify how exposure varies for specific roles within the fire service, culminating in the creation of a JEM.

### Effects Assessment

#### Overall Approach

Effects assessment by the FFCCS includes measurement of biomarkers of effect related to disease pathways and evaluation of cancer and other priority disease outcomes. Biomarkers of effect have included epigenetic markers, such as peripheral blood DNA methylation; microRNA expression; serum anti-müllerian hormone (AMH); urine metabolome; and, in more limited settings, DNA mutations. The use of additional markers is anticipated in the future. Cancer and other priority disease outcomes are evaluated through self-reporting on annual follow-up surveys, which may be supplemented by FFCCS participants’ voluntary participation in the National Firefighter Registry (NFR) for cancer [29] and plans for medical records requests to document cancer diagnosis.

#### Biomarkers of Effect

Cancer is a multistage process with a latency period between exposure and the onset of disease from as short as 5 to >30 years [20]. Molecular biomarkers, including epigenetic alterations, reflect subtle biological changes that occur following exposures that can contribute to subsequent disease development. The epigenome consists of alterations to the genome that do not change the underlying DNA sequence but can be inherited across cell divisions and influence gene regulation. Epigenetic modifications include noncoding RNA expressions, such as microRNAs and DNA methylation. The epigenome controls normal cellular function by regulating gene expression, but aberrant epigenetic modifications contribute to nearly all known disease processes, including cancers [30-33]. While the epigenome differs by cell and tissue type, blood is commonly used as a surrogate tissue in epidemiological studies. Analyzing epigenetics in firefighters’ blood samples and linking these markers to acute or cumulative exposures can reveal subclinical changes that may increase cancer risk.

AMH is a marker of functional ovarian reserve routinely tested during fertility treatments and a proxy for reproductive health, given its association with oocyte yield, infertility diagnosis, time of menopause, miscarriage, and preterm birth. AMH levels steadily decline with age to mirror the decline in the number of oocytes [34]. AMH is, therefore, used for clinically monitoring natural and iatrogenic declines in ovarian function [34,35]. Adverse mental health conditions, including posttraumatic stress disorder and anxiety, have been shown to influence fertility [36] and AMH levels [37]. In addition, AMH levels are susceptible to alterations by inhaled exposures, including smoking and burning fuel for heating or cooking [38]. This supports previous research showing that smoking increases the risk of infertility [39] and earlier age at menopause [40]. Smoking and biomass fuel burning result in similar combustion products, including PAHs, and PAH exposure has been shown to decrease AMH levels [41].

The metabolome can serve as a surrogate composite measure of response to stressor and environmental exposure profiles and may elucidate mechanisms linking exposures to disease. Metabolomic profiling systematically measures thousands of exogenous and endogenous metabolites and can provide key insight into subtle signatures of cancer and other diseases, often before they become clinically apparent [42]. In addition to serving as early biomarkers of risk, prospective studies of metabolites may offer further advantages by identifying biological pathways and metabolites that may themselves be modifiable, either through dietary or other behavioral changes or enhancements to occupational protections. Differential urinary metabolites have been identified when comparing patients with and without breast cancer [43], prostate cancer [44,45], and bladder cancer [46], the latter including those associated with phenylacetate, propanoate, fatty acid, pyruvate, arginine, glycine, and serine metabolism, as well as bile acid biosynthesis. An untargeted metabolomics study of patients with prediagnostic breast cancer found that syringol (a biomarker of wood smoke) was predictive of future breast cancer incidence [47]. In our previous study of metabolomic changes in municipal firefighters after responding to structural fires, we identified changes in endogenous and exogenous metabolites (including the abovementioned metabolites and syringol) that overlap with cancer risk factors, cancer pathways, and specific cancers [48]. Repeated acute stress from fireground exposures on the biological systems that produce these metabolites may contribute to long-term cancer risk. Evaluation of the specific perturbed metabolites for their ability to predict future cancer is needed and may offer a compelling starting point for interventions. As an example, aryl hydrocarbon receptor activation has been associated with cancer risk [49], and we previously identified that aryl hydrocarbon receptor activity increased in firefighter urine extracts after fighting a fire [50].

#### Cancer and Other Disease Outcomes

Disease outcomes (eg, cancer diagnoses) are evaluated through self-report on the annual follow-up survey, with the potential to add other diseases in the future as requested by the fire service. To validate these self-reported cancer outcomes and capture outcomes that are unable to be obtained through FFCCS survey mechanisms, the FFCCS is in the process of promoting participant cross-enrollment and establishing a linkage with the NFR for cancer. The NFR is a congressionally mandated voluntary registry of firefighters established by NIOSH. NFR participants, including those cross-enrolled in FFCCS, will be followed prospectively for any cancer diagnoses by periodically linking with all state cancer registries across the United States. Enrollment in the NFR is being offered during new FFCCS enrollments, and a consent addendum will be offered to existing FFCCS participants during study follow-up visits. Separately, FFCCS processes are being implemented to request medical records that document cancer diagnosis reported on annual follow-up surveys.

### Reporting Results

The FFCCS measures and reports back to participating firefighters a broad range of hazardous exposures and their effects, including urinary levels of PAH metabolites reflecting combined inhalation, dermal, and ingestion exposures, as well as serum levels of PFAS and AMH. Individual report-back templates are also being developed for DNA methylation epigenetic clocks and silicone wristband exposure monitoring results. These personalized results enable firefighters to better understand their exposures and are critical to reporting information back in a timely manner. In addition, a summarized report without personal identifying information is provided back to the fire department. Newsletters are used to share findings from individual FFCCS substudies and are distributed to all FFCCS participants. Furthermore, the study website [51], contains contact information, information on overall organizational structure, substudies, and publications.

### External Data Requests

Data security is at the forefront of all stages of FFCCS data collection and use. FFCCS study participants have expressed concern about the potential use of data outside uses approved by the FFCCS research team and OPB. Specifically, they worry that the FFCCS data could be used against them by their department, municipality, or other administrative entity should they have a workers’ compensation cancer claim or other adverse event. Therefore, in addition to protections in place by the IRB, the FFCCS has obtained a Certificate of Confidentiality from the National Institutes of Health. The informed consent document explains to participants how their data will only be shared with approved research team members and provides information about the Certificate of Confidentiality. To comply with data-sharing requirements associated with specific federal funds (eg, National Institutes of Health), the FFCCS website contains contact information that can be used for requesting deidentified study information. Requests will be reviewed by the FFCCS OPB for their alignment with the overall FFCCS mission and best fit of current datasets to answer requesters’ research questions. For data limited to specific departments, the external request will also be reviewed through a process determined by the fire service research liaisons of the participating fire departments. The process for requesting biological samples from the biobank for research is currently under development.

## Results

### Enrollment to Date

The FFCCS has enrolled 6200 firefighters from >275 fire departments across 31 states as of September 2024. The demographic and firefighter subgroup characteristics of the participating firefighters are shown in Table 2.

**Table 2:** The Fire Fighter Cancer Cohort Study participants’ demographics and department type (N=6200).

Demographics and department type		Recruits (n=1448, 23.4%), n (%)	Incumbents (n=4752, 76.6%), n (%)	Total, n (%)
**Race or ethnicity**				
	American Indian, Alaska Native, Native Hawaiian, or other Pacific Islander	11 (0.7)	51 (1.1)	62 (1)
	Asian	52 (3.6)	85 (1.8)	137 (2.2)
	Black	80 (5.5)	116 (2.4)	196 (3.2)
	Hispanic	368 (25.4)	769 (16.2)	1137 (18.3)
	White, non-Hispanic	715 (49.4)	3234 (68.1)	3949 (63.7)
	Other or multiple	71 (4.9)	227 (4.7)	298 (4.8)
	Missing	151 (10.4)	270 (5.7)	421 (6.8)
**Sex**				
	Male	1050 (72.5)	3927 (82.6)	4977 (80.3)
	Female	346 (23.9)	756 (15.9)	1102 (17.8)
	Missing	52 (3.6)	69 (1.5)	121 (2)
**Department**				
	Career structural	1302 (89.9)	3106 (65.4)	4408 (71.1)
	Volunteer	11 (0.8)	775 (16.3)	786 (12.7)
	Wildland^a^	126 (8.7)	568 (12)	694 (11.2)
	Airport^a^	18 (1.2)	322 (6.8)	340 (5.5)

^a^Some firefighters (n=28) are both wildland and airport firefighters.

The average age of participating firefighters is 37.8 (SD 11) years, including the age of recruits at enrollment (mean 29.3, SD 5 years) and the age of incumbents at enrollment (mean 40.3, SD 11 years). Of 6200 participants, the top 5 states by current enrollment include California (n=1843, 29.72%), Arizona (n=1278, 20.61%), Massachusetts (n=597, 9.63%), Florida (n=277, 4.48%), and Washington (n=273, 4.4%).

In addition to career municipal firefighters, separate grants have supported the enrollment of firefighter subgroups, including volunteers; trainers; fire investigators; and women, wildland, WUI, and airport firefighters. Of 6200 participants, 1102 (17.8%) are women firefighters, 196 (3.2%) are Black, 1137 (18.3%) are Hispanic, 3949 (63.7%) are White and non-Hispanic, and 298 (4.8%) are multiple or of another race. Career firefighters constitute 71.1% (n=4408) of our study participants, volunteer firefighters 12.7% (n=786), wildland firefighters 11.2% (n=694), and airport firefighters 5.5% (n=340), with some participants (n=28, 0.5%) categorized as both wildland and airport firefighters.

### Biological Sample Collection, Analysis, and Repository

As of September 2024, a total of 11,738 blood samples have been analyzed for serum PFAS concentration by either the New Jersey Department of Health Public Health and Environmental Laboratories, Eurofins, or NCEH within the CDC. Furthermore, 1248 blood samples from women firefighters have been analyzed for AMH by Motive Biosciences (Webster), and 638 urine samples have been analyzed for hydroxylated metabolites of PAHs by the New Jersey Department of Health, NCEH, or the Human Health Exposure Analysis Resource Laboratory.

### Early Study Results

The FFCCS has already expanded our knowledge of firefighter exposures to everyone on the fireground [52], increases in serum levels of PFAS in firefighters compared with the general population and the effects of workplace exposures [27,28,53], the effectiveness of exposure reduction protocols established by the fire service [54], differences in cancer prevention practices in the volunteer fire service [55,56], and cancer risk perceptions among women firefighters [57]. Furthermore, we have found marked reductions in serum AMH levels in female firefighters associated with self-reported clinical diagnoses of anxiety and posttraumatic stress disorder, as well as characterizations of stress and posttraumatic stress disorder [58-60]. Epigenetic findings in the FFCCS to date include the identification of DNA methylation loci and microRNA that differ in incumbent and recruit municipal firefighters [61,62]. When following up recruits over time (approximately 2 years of employment), microRNA and DNA methylation change, including in cancer-related genes [26,63]. We have also reported differences in epigenetic age, a biomarker of biological aging, by serum levels of PFAS and when comparing recruits with incumbents [64,65], as well as differential methylation of prostate cancer genes in association with years of firefighting and serum levels of PFAS [66].

In addition, we have compared metabolites in the urine of firefighters at baseline and after a fire and observed broad changes, including altered excretion of indole compounds, uremic toxins, several amino acids, and hormones and alteration of the vitamin B3 (nicotinate and nicotinamide) pathway [48]. We hypothesized that if disruption of uremic pathways and increased excretion of uremic toxins are experienced regularly, then multiple acute hits to this pathway may mediate firefighter’s increased risk for urinary tract cancers. Moreover, we used the untargeted metabolomics results for hypothesis generation; for instance, we observed some overlap among elevated metabolites after fires with metabolites that have been implicated in Alzheimer disease and related dementias.

## Discussion

### Overview

The FFCCS provides a framework for community-engaged prospective evaluation of firefighter exposures, mechanisms of toxicity, and cancer outcomes through the integration of survey, biological, and exposure data. This work adds to the existing growing body of literature from firefighter cohorts around the world, including additional cohorts within the United States and other global cohorts in Korea, Australia, and other regions of the world [67,68]. These cohorts have examined markers of cardiovascular health, environmental exposures, the impact of disasters such as 9/11, and cancer incidence [69-71]. The FFCCS has contributed to scientific knowledge within the fire service and beyond, and with continued expansion and participant follow-up, the potential to identify important exposures, toxic mechanisms, effective exposure reduction, and chronic disease preventive activities will increase markedly. Additional health outcomes prioritized by the fire service and researchers, such as adverse reproductive effects, are also being evaluated.

### Strengths

The strengths of the FFCCS include its ongoing engagement of the firefighter community in research, the multicenter prospective approach permitting evaluation of different firefighter subgroups, regional exposures and fire service practices, the inclusion of recruit and incumbent firefighters, and the establishment of a biobank. Participation of firefighters in all phases of the research helps ensure that the research questions address fire service concerns and that the research results can be used to inform fire service policies. Differences in cancer rates by geographic region have been noted in previous US studies of firefighters [2,3], and very little is known about cancer rates in US firefighters outside of male career structural firefighters, emphasizing the need for the multicenter approach to establishing a firefighter cancer cohort. This study has effectively prioritized the inclusion of women, with female firefighters making up >17% (1102/6200) of the participants despite women comprising only 9% of the overall US firefighting population [14]. The inclusion of recruit firefighters before live-fire exposure provides a comparison group to help identify changes specific to firefighting, adjusting for age differences between the 2 groups. Furthermore, the prospective nature of the study and collection of biomarkers of exposure and effect samples before the establishment of cancer is an essential strength of the study, along with the establishment of a biobank enabling future analyses on stored samples.

### Limitations

The limitations of the FFCCS include the potential for self-selection bias; the lack of a defined nonfirefighter longitudinal comparison group; some types of self-reported survey information, such as occupational exposure history; and the lack of long-term funding. As participation rates vary among groups offered enrollment in the study, with enrollment among recruits generally >80% and incumbent enrollment generally <50%, there may be differences in exposures and biomarkers of effect when comparing firefighters choosing and not choosing to participate in the FFCCS. Although we adjusted for age, some longitudinal changes in measured biomarkers of effect may be due to age alone rather than occupational exposure. In addition, although firefighters generally tend to remain in the same department, only participants continuing to serve as active firefighters will be kept in the biomarker collection part of the study. Results from this study may also be impacted by the healthy worker effect [69,72]. The study includes self-reported survey information, which may be subject to recall bias. The lack of long-term funding may limit our ability to conduct regular follow-up evaluations, including blood and urine collection from all study participants outside of currently funded projects.

### Future Directions

The FFCCS will continue to expand as we work toward reaching our target enrollment of 10,000 firefighters, focusing on the goal of advancing firefighter cancer control and prevention while evaluating other health conditions. Specifically, we will continue to enroll and categorize exposures among subgroups, such as women firefighters, understudied racial and ethnic groups, wildland firefighters, WUI firefighters, and firefighters responding to high-risk events. Expansion within these subgroups is critical to accomplishing the mission of the FFCCS. In addition, study findings will continue to be regularly communicated to participants and our fire service partners as the study progresses.

### Conclusions

The establishment of the FFCCS, in collaboration with the fire service community, has provided a unique research platform to advance the understanding of firefighter exposures, help identify biomarkers of carcinogenic effect and cancer risk, and provide a setting for the observational evaluation of the effects of exposure reduction and wellness activities carried out by the fire service.

## Abbreviations

AMH: anti-müllerian hormone
CAPS: Firefighter Cancer Assessment and Prevention Study
CDC: Centers for Disease Control and Prevention
EDTA: ethylenediaminetetraacetic acid
FFCCS: Fire Fighter Cancer Cohort Study
IARC: International Agency for Research on Cancer
IRB: institutional review board
JEM: job exposure matrix
NCEH: National Center for Environmental Health
NFORS: National Fire Operations Reporting System
NFR: National Firefighter Registry
NIOSH: National Institute for Occupational Safety and Health
OPB: oversight and planning board
PAH: polycyclic aromatic hydrocarbon
PER: Personal Exposure Reporter
PFAS: per- and polyfluoroalkyl substances
PI: principal investigator
REDCap: Research Electronic Data Capture
VOC: volatile organic compound
WUI: wildland-urban interface
